# Effect of Seasonal Influenza Vaccines on Avian Influenza A(H5N1) Clade 2.3.4.4b Virus Infection in Ferrets

**DOI:** 10.3201/eid3110.250668

**Published:** 2025-10

**Authors:** Xiangjie Sun, Jeeva Subbiah, Jessica A. Belser, Nicole Brock, Shane Gansebom, Zhu-Nan Li, Yu-Jin Jung, Feng Liu, Terrence M. Tumpey, Taronna R. Maines, Min Z. Levine, Ian A. York

**Affiliations:** Centers for Disease Control and Prevention, Atlanta, Georgia, USA (X. Sun, J.A. Belser, N. Brock, Z.-N. Li, Y.-J. Jung, F. Liu, T.M. Tumpey, T.R. Maines, M.Z. Levine, I.A. York); Salt Tribe Incorporated-Federal/Cherokee Nation Operational Solutions, Atlanta (J. Subbiah, S. Gansebom)

**Keywords:** Influenza, vaccines, viruses, avian influenza, H5N1, ferrets, clade 2.3.4.4b, United States

## Abstract

Highly pathogenic avian influenza A(H5N1) clade 2.3.4.4b viruses have infected >1,000 herds of dairy cattle and hundreds of poultry flocks in the United States since the beginning of 2024. Seventy human cases have been reported during that period, mainly through occupational exposure. Although prior influenza A(H1N1)pdm09 virus infection has been shown to confer protection against influenza A(H5N1) clade 2.3.4.4b virus infection in the ferret model, it remains unclear if influenza vaccines, known to elicit a less potent and narrower cross-reactive immune response, can achieve a similar effect. In this article, we demonstrate that immunization with commercially available human seasonal influenza vaccines also confers partial protection against disease caused by H5N1 clade 2.3.4.4b virus in ferrets, which is partially associated with the presence of cross-reactive antibodies targeting H5N1 virus antigens.

Human seasonal influenza A viruses (IAVs) are endemic in humans, causing 9.3–41 million cases, 120,000–710,000 hospitalizations, and 6,300–52,000 deaths annually in the United States (*1*). Humans might also be infected by IAVs with zoonotic potential circulating in many species, including wild and domestic birds and mammals. Although human infections with zoonotic IAVs are sporadic, the viruses have the potential for sustained transmission in humans, potentially leading to global pandemics with relatively high morbidity and mortality rates, as seen in 1918, 1957, 1968, and 2009 (*2*,*3*).

Most adult humans have been repeatedly infected with or vaccinated against multiple seasonal influenza A and B viruses and have antibody and T-cell responses against IAV components, including the major surface glycoproteins hemagglutinin (HA) and neuraminidase (NA). Although antigenic drift in HA and NA enables IAVs to reinfect persons despite immunity, immune responses that are even partially matched to the infecting virus are believed to confer some protection against disease (*4*). Zoonotic strains of IAV often express HA subtypes that are distantly related to endemic seasonal strains, and the general population typically exhibits low levels of or no HA head binding antibodies to potential pandemic viruses (*5*; Z.-N. Li et al., unpub. data, https://www.medrxiv.org/content/10.1101/2025.03.30.25323419v1).

Since the first documented human infection with highly pathogenic avian influenza (HPAI) A(H5N1) virus was reported in China and Hong Kong in 1997, this virus subtype has remained a major public health concern because of its ability to cross species barriers and infect humans (*6**,**7*), often causing severe or fatal disease. Originally found in Asia, HPAI H5N1 viruses have spread widely in birds, likely because of wild bird migration routes. HPAI H5N1 viruses have undergone extensive evolution and countless reassortments, leading to many different clades on the basis of HA sequence. Of those clades, clade 2.3.4.4b has emerged as the most widespread in recent years. In 2021, HPAI H5N1clade 2.3.4.4b viruses began to spread widely across the Americas (*8**,**9*), continuing to diversify, and leading to outbreaks in commercial and backyard poultry across the United States. In 2024 and 2025, clade 2.3.4.4b virus of the B3.13, D1.1, and D1.3 genotypes caused infections in dairy cows and poultry in North America (*10**,**11*). As of April 10, 2025, there have been 70 H5N1 human infections with those clades reported and 1 death (*12*). Of note, in contrast to the severe disease that has historically been associated with H5N1 virus infections outside of the United States (*13**,**14*), most of the human infections with the clade 2.3.4.4b B3.13 and D1.1 genotypes have been relatively mild (*15**,**16*), manifesting primarily as conjunctivitis and mild respiratory symptoms.

Ferrets are a small-animal model used for human IAV infection because they often show similar clinical manifestations and severity of infection with IAV strains, without needing to adapt the virus to ferrets (*17*). However, in contrast to the mild disease in humans, ferrets infected with clade 2.3.4.4b viruses have typically shown severe or lethal infections (*18**,**19*). One possible explanation for this contrasting severity is that humans might have cross-protective immunity to this clade of viruses because of prior infection with human seasonal strains of IAV, whereas the laboratory ferrets used are immunologically naive to all strains of influenza. Several groups have demonstrated that prior infection of ferrets with human influenza A(H1N1)pdm09 (pH1N1) virus confers major cross-protection against 2.3.4.4b B3.13 viruses (*20*,*21*; P.H. Brigleb et al., unpub. data, https://www.biorxiv.org/content/10.1101/2024.10.23.619695v1). However, the mechanisms underlying that cross-protection remain unclear.

Although vaccination against human seasonal IAV remains the most effective way of protecting against seasonal influenza virus infection, it is unclear whether seasonal influenza vaccine immunity could confer cross-protection against H5N1 viruses. In this article, we report the results of testing the ability of several human seasonal vaccines to protect ferrets against disease induced by clade 2.3.4.4b B3.13 IAV infection.

## Materials and Methods

### Vaccines, Ferrets, and Viruses

The vaccines used in this study were for the 2024–25 Northern Hemisphere influenza season. The vaccines were the egg-based trivalent inactivated influenza vaccine (IIV3) Fluarix (GlaxoSmithKline Biologicals, https://www.gsk.com), the live attenuated influenza vaccine (LAIV) FluMist (AstraZeneca, https://www.astrazeneca.com), and the recombinant hemagglutinin vaccine (RIV) Flublok (Sanofi, https://www.sanofi.com). Six-month-old male fitch ferrets (Triple F Farms, https://www.tripleffarmsresearch.com) were housed in Duo-Flo Bioclean units (Lab Products, Inc., https://labproductsllc.com) at Centers for Disease Control and Prevention (CDC) animal facilities. Animal studies were approved by CDC’s Institutional Animal Care and Use Committee and were conducted in American Association for Accreditation for Laboratory Animal Care accredited animal facilities. 

We inoculated ferrets with the manufacturers’ recommended human doses of 0.5 mL IIV3 intramuscularly (IM) (containing 15 µg HA from each virus strain), 0.5 mL of RIV IM (containing 45 µg HA of each strain), or 0.2 mL of LAIV intranasally (IN) (containing 10^6.5–7.5^ fluorescent focus units of live attenuated influenza virus reassortant of each strain). IIV3 also contains NA from each virus, although the quantity of NA included is not regulated and varies between virus strains (*22*). The RIV does not contain NA.

We challenged ferrets with A/Michigan/90/2024 A(H5N1) (MI/24) (GISAID [https://gisaid.org] accession no. EPI_ISL_19162802), a clade 2.3.4.4b, B3.13 genotype virus isolated from a human experiencing mild disease (*23*), which has been shown to cause moderate, nonfatal disease in laboratory ferrets (*24*). We conducted all HPAI preparation and animal infection experiments in Biosafety Level 3 containment laboratories, including enhancements regulated by the US Department of Agriculture and the Federal Select Agent Program.

### Ferret Immunization and Challenge

We confirmed ferrets to be initially serologically negative for current circulating human influenza viruses (pH1N1, H3N2, and influenza B) by hemagglutination inhibition assay (HAI). We immunized groups of 6 ferrets 2 times at 3-week intervals IM with RIV or with phosphate-buffered saline (PBS) and IN with LAIV. The IIV3 group did not achieve serologic titers that are correlated with protection after 2 immunizations and therefore received a third IM immunization, 3 weeks apart. We used a separate mock-immunized control group for those animals. Because the outcomes of infection in both mock-immunized groups were statistically similar (body weight, p = 0.093; temperature, p = 0.96; viral shedding, p = 0.64), we have presented them as 1 merged group. We challenged ferrets by IN inoculation anesthesia, with 10^6^ plaque-forming unit (PFU) MI/24 diluted in PBS in 1 mL volume, 24–25 days after final immunization. We monitored ferrets daily for clinical signs of infection, including body weight, temperature, and lethargy (*25**,**26*). We calculated the relative inactivity index for each group on the basis of a previously published scoring system (*27*). We collected nasal wash samples for virus titers at 1–4, 6, and 8 days postinoculation (DPI).

### Hemagglutination Inhibition Assay

We conducted HAI assays by using a World Health Organization kit (International Reagent Resource, https://www.internationalreagentresource.org) against A/Victoria/2570/2019 (H1N1)pdm09 and A/Delaware/01/2021(H3N2), as previously described (*28*). We treated ferret serum with a receptor-destroying enzyme (Denka Seiken, https://www.denka.co.jp). We then serially diluted the treated serum samples 2-fold with PBS, mixed with virus diluted to 8 hemagglutination units; we then incubated the samples at room temperature with 0.5% turkey red blood cells in PBS. We conducted HAI against MI/24 virus by using 1% horse red blood cells in PBS as previously described (*29*). We expressed HAI titers as the reciprocal of the highest dilution of the serum samples completely inhibiting hemagglutination.

### Enzyme-Linked Lectin Assay

We assessed the neuraminidase inhibition (NAI) activity of ferret immune serum against A/Wisconsin/67/2022 A(H1N1)pdm09, A/Massachusetts/18/2022 A(H3N2), and A/Texas/37/2024 A(H5N1) (clade 2.3.4.4b, genotype B3.13) by using an enzyme-linked lectin assay (ELLA) with a fetuin-based method, as previously described (*30*). We added serum and virus to a 96-well plate precoated with 25 μg/mL of fetuin (Sigma-Aldrich, https://www.sigmaaldrich.com), followed by overnight incubation at 37°C. We then quantified NAI activity by using horseradish peroxide-labeled peanut lectin with O-phenylenediamine dihydrochloride substrate. We calculated the percentage of inhibition by using the formula 100 × optical density (OD) virus only – OD test sample)/OD virus only. We assigned samples below the limit of detection a titer of 10.

### Multiplex Influenza Antibody Detection Assay

We performed a high-throughput multiplex influenza antibody detection assay (MIADA), as described previously (*31*). We included HA globular head, HA ectodomain, HA stalk, NA ectodomain, and nucleoprotein (NP) antigens from IAVs and the HA globular head from influenza B viruses (Appendix Table). In a 96-well black-wall plate, we added 50 µL of microsphere suspension (2,000 microspheres/antigen) to each well in assay buffer. We added diluted ferret serum (1:200) in duplicate. We incubated the plates for 60 minutes, washed, and then added 100 µL of protein A–phycoerythrin conjugate. After another 60-minute incubation and washing, we read the plates by using a Bio-Plex MAGPIX Multiplex Reader (Bio-Rad Laboratories, https://www.bio-rad.com). We calculated median fluorescence intensities by using GraphPad version 7.05 (GraphPad Software Inc., https://www.graphpad.com).

### Virus Titration

At 3 DPI, we euthanized a subset of ferrets (n = 3/group) and collected tissue samples from the upper respiratory tract (nasal turbinate, ethmoid turbinate, soft palate, and trachea), lower respiratory tract (lung), gastrointestinal tract (intestine), olfactory bulb, and brain tissues. We then determined viral loads in the tissues and nasal wash (NW) by using standard plaque formation assays in MDCK cells (limit of detection is 10 PFU/mL or PFU/g of tissues).

### Statistical Analysis

We analyzed body weights and NW titers with a linear mixed model with repeated measures by using R version 4.4.0 with lmerTest version 3.1.3 (The R Project for Statistical Computing, https://www.r-project.org). We analyzed the tissue virus titers and antibody titers by using analysis of variance with Dunnett’s posthoc test for multiple comparisons by using GraphPad version 7.05.

## Results

### Serologic Response to Vaccination

**Figure 1:**
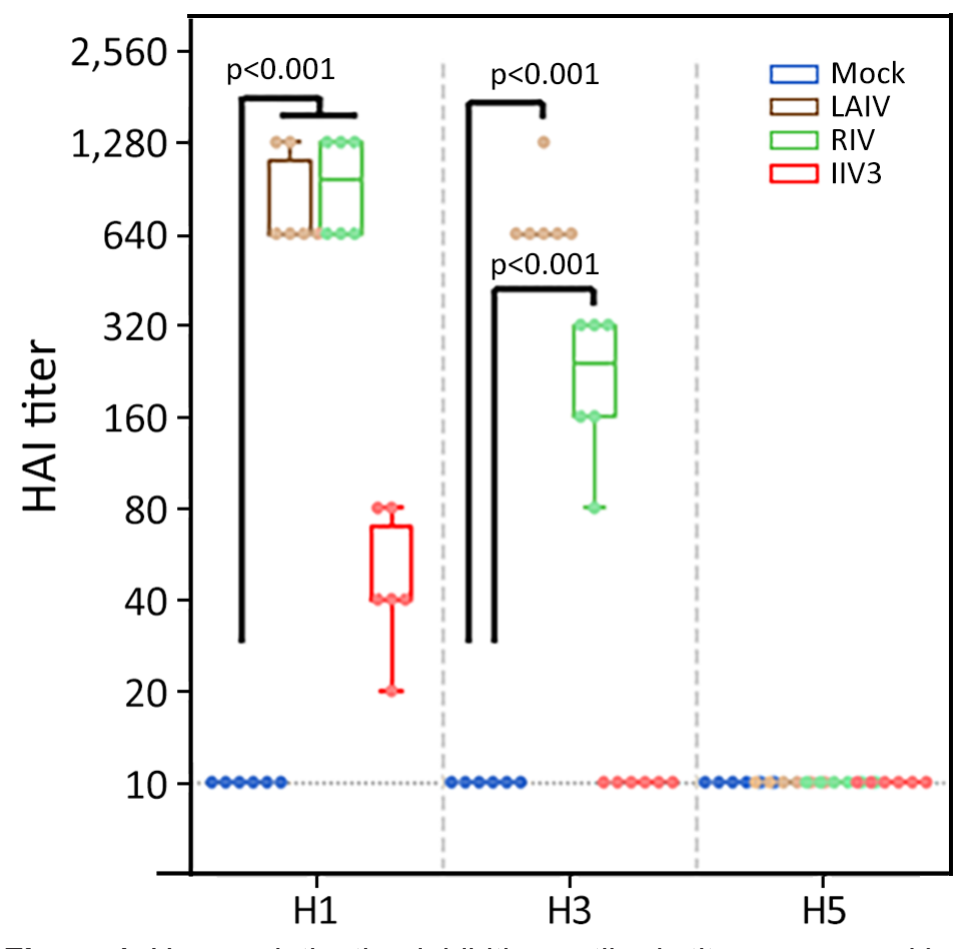
Hemagglutinin-inhibiting antibody titers measured by using HAI in study of the effect of seasonal influenza vaccines on influenza A(H5N1) clade 2.3.4.4b virus infection in ferrets. n = 6 ferrets per vaccinated group, n = 12 ferrets in the mock vaccinated group. Colored dots indicate individual values, horizontal lines within boxes represent geometric mean titers, box tops and bottoms indicate interquartile ranges, and error bars indicate limits of the distribution. Dotted gray horizontal line indicates limit of detection. H1, A/Victoria/2570/2019(H1N1)pdm09; H3, A/Delaware/01/2021(H3N2); H5, A/Michigan/90/2024/(H5N1); HAI, hemagglutination inhibition assay; IIV3, Fluarix trivalent inactivated influenza vaccine (GlaxoSmithKline Biologicals, https://www.gsk.com); LAIV, FluMist live attenuated influenza vaccine (AstraZeneca, https://www.astrazeneca.com); RIV, Flublok recombinant influenza vaccine (Sanofi, https://www.sanofi.com).

Before challenge with A(H5N1), we evaluated the antibody responses against HA and NA antigens of seasonal IAV A/Wisconsin/67/2022 A(H1N1)pdm09, A/Massachusetts/18/2022 A(H3N2), and clade 2.3.4.4b A(H5N1) virus (MI/24 or TX/22) in the serum of vaccinated ferrets and the control group by using HAI assay or ELLA. Among the 3 vaccinated groups, 2 doses of LAIV or RIV vaccination elicited robust HAI titers against H1 HA (Table 1; Figure 1), whereas the IIV3 vaccinated group exhibited much lower titers even after a second vaccine booster (Table 1; Figure 1). HAI titers against the H3N2 virus were generally lower than those against the pH1N1 virus, and IIV3 groups had no detectable HAI titers against H3N2 virus. The HAI antibody titer against MI/24 virus was below detectable levels in all 3 vaccinated groups (Table 1; Figure 1).

**Table 1:** Serologic response to vaccination in ferrets in study of the effect of seasonal human influenza vaccination on pathogenesis of human influenza A(H5N1) clade 2.3.4.4b virus in the ferret model*

Vaccine group	IAV lineage	Vaccine strain	Prechallenge HAI GMT	Prechallenge ELLA GMT
LAIV	A(H1N1)pdm09	NO/22	806 (606–1074)	320 (236–434)
	A(H3N2)	TH/22	718 (573–901)	71 (54–95)
	A(H5N1)	NA	<10	<10
RIV	A(H1N1)pdm09	WV/22	905 (668–1227)	<10
	A(H3N2)	MA/22	202 (128–317)	<10
	A(H5N1)	NA	<10	<10
IIV3	A(H1N1)pdm09	VIC/22	45 (30–68)	<10
	A(H3N2)	TH/22	<10	<10
	A(H5N1)	NA	<10	18.8 (13.0–27.3)
MOCK	A(H1N1)pdm09	NA	<10	<10
	A(H3N2)	NA	<10	<10
	A(H5N1)	NA	<10	<10

*Values in parentheses are 95% CIs. n = 6 ferrets per vaccine group, n = 12 ferrets in the mock group. The vaccines contained antigens recommended for the 2024–25 Northern Hemisphere influenza season. ELLA, enzyme-linked lectin assay; GMT, geometric mean titer; HAI, hemagglutination inhibition assay; IAV, influenza A virus; IIV3, Fluarix trivalent inactivated influenza vaccine (GlaxoSmithKline Biologicals, https://www.gsk.com); LAIV, FluMist live attenuated influenza vaccine (AstraZeneca, https://www.astrazeneca.com); MA/22, A/Massachusetts/18/2022 A(H3N2); NA, not available; NO/22, A/Norway/31694/2022 A(H1N1)pdm09; RIV, Flublok vaccine (Sanofi, https://www.sanofi.com); TH/22, A/Thailand/8/2022 A(H3N2); WV/22, A/West Virginia/30/2022 A(H1N1)pdm09; VIC/22, A/Victoria/4897/2022 A(H1N1)pdm09.

**Figure 2:**
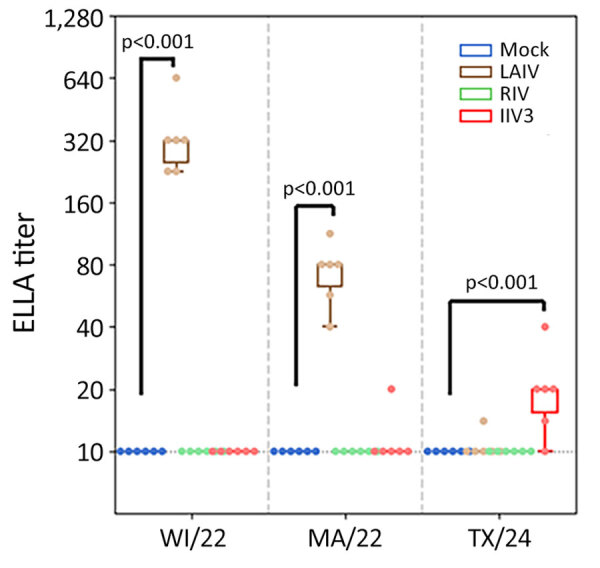
Neuraminidase-inhibiting antibody titers measured by using ELLA in study of the effect of seasonal influenza vaccines on influenza A(H5N1) clade 2.3.4.4b virus infection in ferrets. n = 6 ferrets per the vaccinated group, n = 12 ferrets in the mock vaccinated group. Colored dots indicate individual values, horizontal lines within boxes represent geometric mean titers, box tops and bottoms indicate interquartile ranges, and error bars indicate limits of the distribution. Dotted gray horizontal line indicates limit of detection. ELLA, enzyme-linked lectin assay; IIV3, Fluarix trivalent inactivated influenza vaccine (GlaxoSmithKline Biologicals, https://www.gsk.com); LAIV, FluMist live attenuated influenza vaccine (AstraZeneca, https://www.astrazeneca.com); MA/22, A/Massachusetts/18/2022(H3N2); RIV, Flublok recombinant influenza vaccine (Sanofi, https://www.sanofi.com); TX/24, A/Texas/37/2024(H5N1); WI/22, A/Wisconsin/67/2022(H1N1pdm09).

In naive ferrets immunized with LAIV, the production of functional NAI antibodies against N1 from pH1N1 virus was readily detected by ELLA (geometric mean titer [GMT] of 319.9 [95% CI 236.0–433.6]), whereas those immunized with IIV3 had an undetectable NAI antibody response to N1 from pH1N1 virus (Figure 2). Ferrets immunized with LAIV had a detectable antibody response against N2 NA, whereas those immunized with IIV3 did not. IIV3-immunized ferrets had detectable responses to N1 from H5N1 (GMT 18.8 [95% CI 13.0–27.3]; p = 0.005 compared with mock-immunized ferrets). Titers against N1 from H5N1 exceeded the limit of detection of 10 in only 1 of 6 LAIV-immunized animals (Figure 2). Because NA is not present in RIV, no anti-NA response was detected in ferrets immunized with this vaccine.

**Figure 3:**
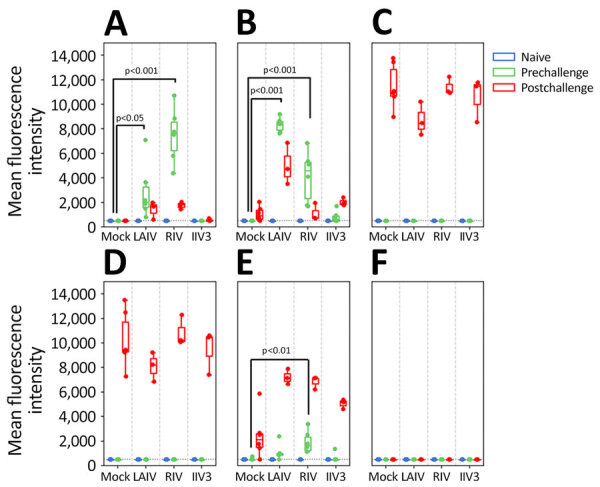
Hemagglutinin-binding antibody titers measured by using multiplex influenza antibody detection assay in study of the effect of seasonal influenza vaccines on influenza A(H5N1) clade 2.3.4.4b virus infection in ferrets. n = 6 ferrets per vaccinated group, n = 12 ferrets in the mock vaccinated group. A) Globular head of hemagglutinin H1 from influenza A/Wisconsin/67/2022(H1N1pdm09). B) Globular head of hemagglutinin H3 from A/Massachusetts/18/2022(H3N2). C) Globular head of hemagglutinin H5 from A/Texas/37/2024(H5N1). D) Ectodomain of hemagglutinin H5 from A/Texas/37/2024(H5N1). E) Stalk of hemagglutinin H1 from A/Michigan/45/2015(H1N1pdm09). F) Stalk of hemagglutinin H3 from A/Singapore/INFIMH-16-0019/2016(H3N2). Colored dots indicate individual values, horizontal lines within boxes represent geometric mean titers, box tops and bottoms indicate interquartile ranges, and error bars indicate limits of the distribution. Dotted gray horizontal line indicates limit of detection. IIV3, Fluarix trivalent inactivated influenza vaccine (GlaxoSmithKline Biologicals, https://www.gsk.com); LAIV, FluMist live attenuated influenza vaccine (AstraZeneca, https://www.astrazeneca.com); RIV, Flublok recombinant influenza vaccine (Sanofi, https://www.sanofi.com).

**Figure 4:**
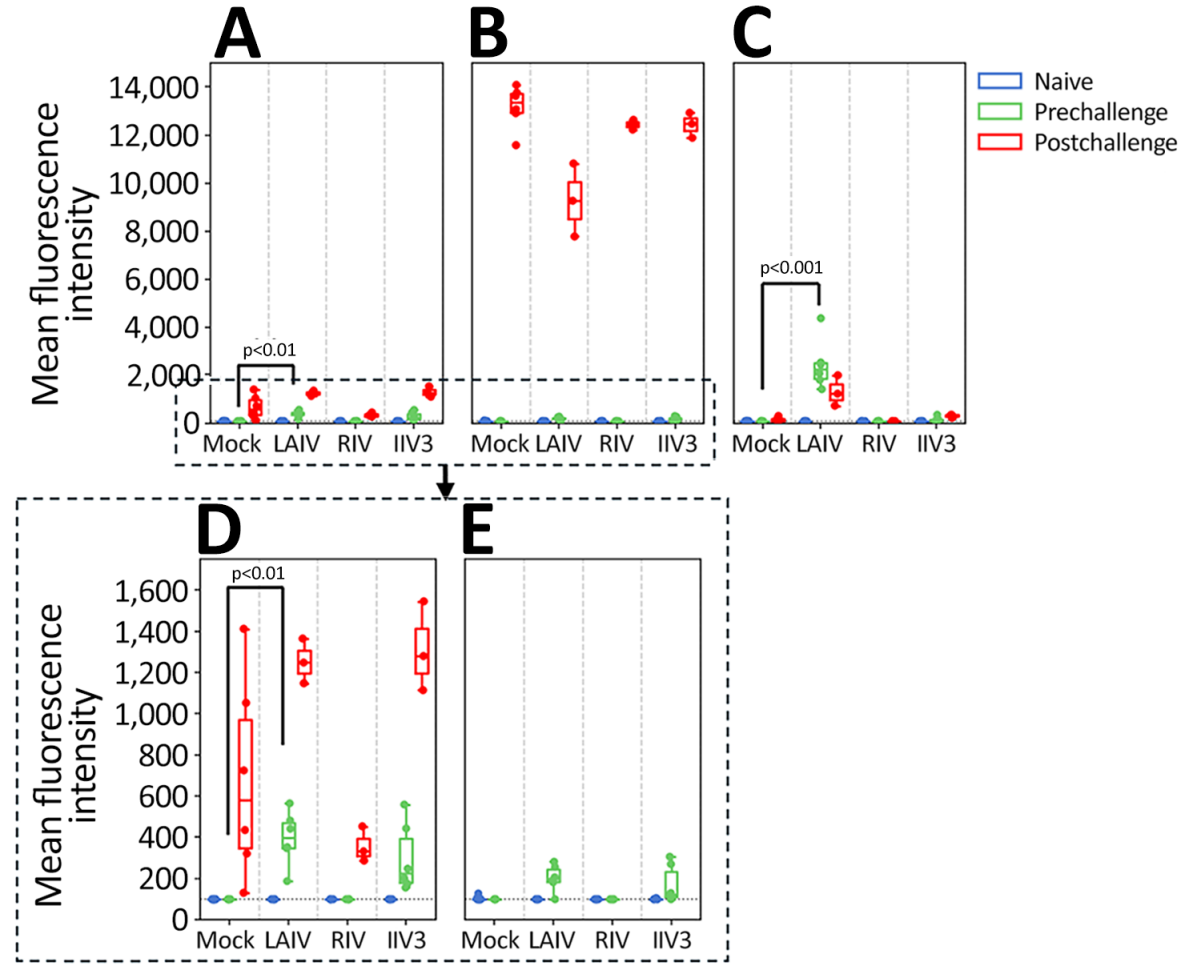
Neuraminidase antibody titers measured by using multiplex influenza antibody detection assay in study of the effect of seasonal influenza vaccines on influenza A(H5N1) clade 2.3.4.4b virus infection in ferrets. n = 6 ferrets per vaccinated group, n = 12 ferrets in the mock vaccinated group. A) Neuraminidase N1 from A/Wisconsin/67/2022(H1N1pdm09). B) Neuraminidase N1 from A/Texas/37/2024(H5N1). C) Neuraminidase N2 from A/Massachusetts/18/2022(H3N2). D) Expanded view of neuraminidase N1 from A/Wisconsin/67/2022(H1N1pdm09) to more clearly show the cross-reactive responses postvaccination. E) Expanded view of neuraminidase N1 from A/Texas/37/2024(H5N1) to more clearly show the cross-reactive responses postvaccination. Colored dots indicate individual values, horizontal lines within boxes represent geometric mean titers, box tops and bottoms indicate interquartile ranges, and error bars indicate limits of the distribution. Dotted gray horizontal line indicates limit of detection. IIV3, Fluarix trivalent inactivated influenza vaccine (GlaxoSmithKline Biologicals, https://www.gsk.com); LAIV, FluMist live attenuated influenza vaccine (AstraZeneca, https://www.astrazeneca.com); RIV, Flublok recombinant influenza vaccine (Sanofi, https://www.sanofi.com).

**Figure 5:**
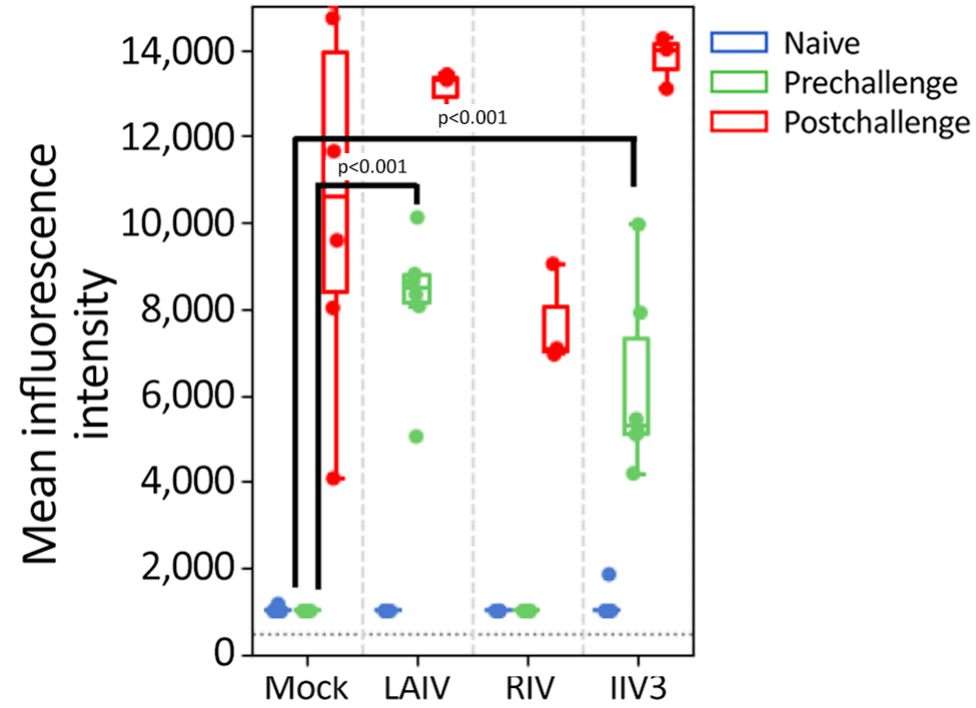
Nucleoprotein antibody titers for A/Brisbane/10/2007(H3N2) virus measured by using multiplex influenza antibody detection assay in study of the effect of seasonal influenza vaccines on influenza A(H5N1) clade 2.3.4.4b virus infection in ferrets. n = 6 ferrets per vaccinated group, n = 12 ferrets in the mock vaccinated group. Colored dots indicate individual values, horizontal lines within boxes represent geometric mean titers, box tops and bottoms indicate interquartile ranges, and error bars indicate limits of the distribution. Dotted gray horizontal line indicates limit of detection. IIV3, Fluarix trivalent inactivated influenza vaccine (GlaxoSmithKline Biologicals, https://www.gsk.com); LAIV, FluMist live attenuated influenza vaccine (AstraZeneca, https://www.astrazeneca.com); RIV, Flublok recombinant influenza vaccine (Sanofi, https://www.sanofi.com).

In addition, we tested ferret serum from prevaccination and prechallenge and postchallenge time points in each group with MIADA to assess levels of binding antibodies against a range of antigens, including HA globular head, ectodomain, stalk, and NA ectodomain of pH1N1, H3N2, H5N1 viruses, and HA globular heads of influenza B virus and NP antigen. In agreement with HAI and NAI antibody titers, antibodies binding the H1 and H3 HA globular head and N1 and N2 NA ectodomains were readily detected in LAIV-immunized ferrets (Figures 3, 4). RIV-immunized ferrets also had antibodies targeting H1 and H3 HA. In addition, LAIV and RIV groups elicited detectable binding antibodies targeting group 1 (H1), but not group 2 (H3), HA stalk regions after 2 vaccinations, whereas these antibodies were not detected in the IIV3 group even after 3 vaccinations (Figure 3). Three of 6 ferrets immunized with LAIV and 2 of 6 immunized with IIV3 had detectable levels of cross-reactive binding antibodies to N1 of H5N1 (LAIV, GMT 223.3 [95% CI 194.5–252.0]; p = 0.37 relative to mock-immunized ferrets; IIV3, GMT 228.5 [95% CI 190.8–266.2]; p = 0.23) (Figure 4). Ferrets immunized with LAIV and IIV3 also possessed detectable NP binding antibody before challenge, which markedly increased postchallenge (Figure 5). As expected, RIV-immunized animals did not develop antibodies against NP or cross-reactive H5N1 NA antibodies before challenge (Figures 4, 5). Antigens used in the MIADA are provided (Appendix Table), and the results of the assay for all 27 antigens are also provided (Appendix Figure).

### Vaccine Protection Against H5N1 Disease

**Figure 6:**
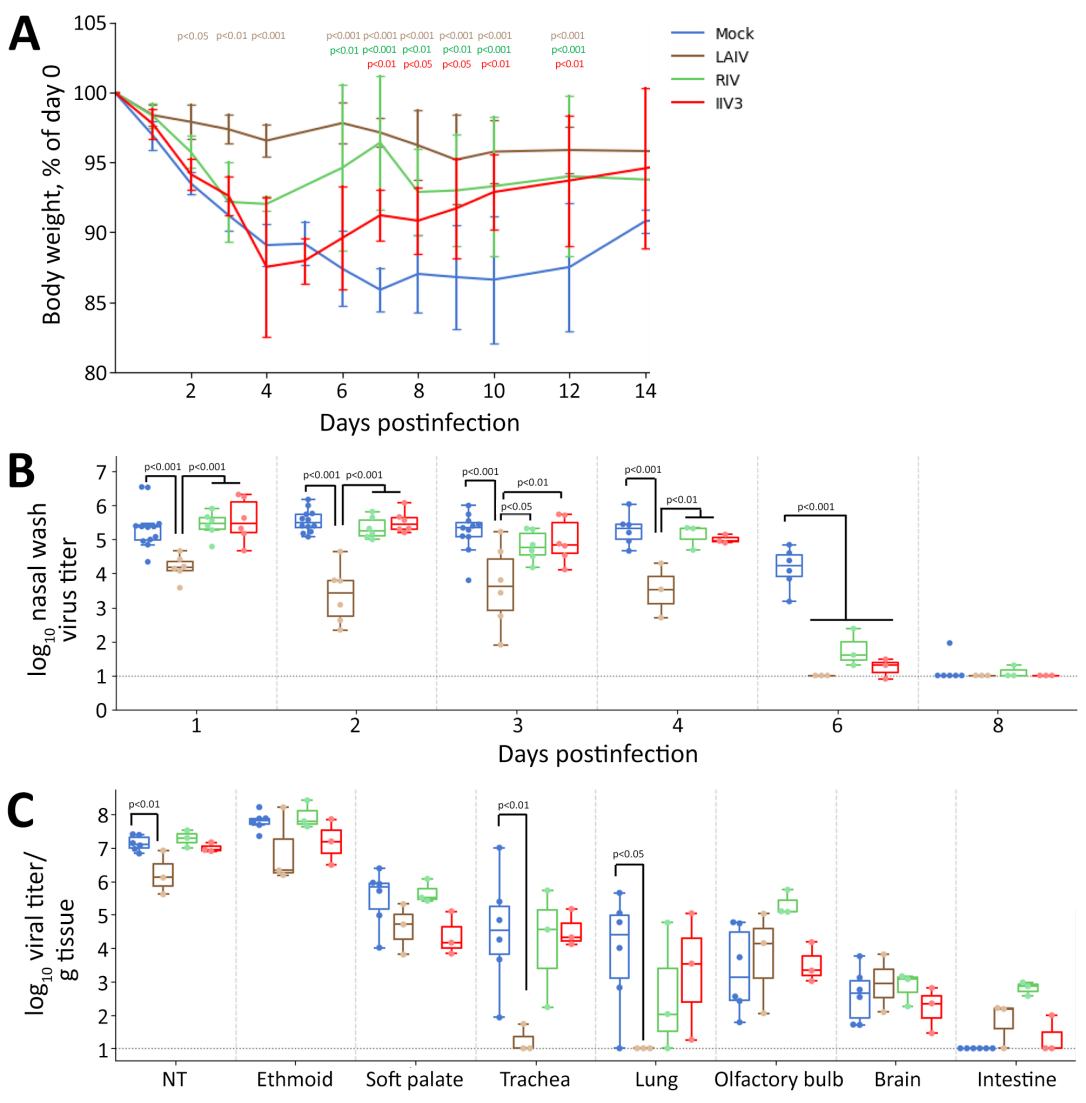
Body weight and influenza A(H5N1) clade 2.3.4.4b viral titers from nasal wash and tissue samples collected during study of the effect of seasonal influenza vaccines on clade 2.3.4.4b H5N1 virus infection in ferrets. n = 6 ferrets per vaccinated group, n = 12 ferrets in the mock vaccinated group. A) Body weights were monitored for 14 days postinfection and scaled to each ferret’s weight on day 0. We assessed p values relative to the mock-infected group by using a linear mixed model with repeated measures in R with lmerTest version 3.1.3 (The R Project for Statistical Computing, https://www.r-project.org); p values are indicated in colors matching the relevant body weight traces. Lines are plotted as means ± SDs. B) We collected nasal washes with phosphate buffered saline and measured the amount of virus by standard plaque formation assays. We assessed p values by using a linear mixed model with repeated measures in R with lmerTest version 3.1.3. C) Three days after infection, we euthanized 3 ferrets per immunized group and 6 ferrets in the mock control group. We measured the amount of virus present in each tissue sample by standard plaque formation assay and assessed p values relative to the mock-infected group. In panels B and C, colored dots indicate individual values, horizontal lines within boxes represent geometric mean titers, box tops and bottoms indicate interquartile ranges, and error bars indicate limits of the distribution. Dotted gray horizontal line indicates limit of detection. IIV3, Fluarix trivalent inactivated influenza vaccine (GlaxoSmithKline Biologicals, https://www.gsk.com); LAIV, FluMist live attenuated influenza vaccine (AstraZeneca, https://www.astrazeneca.com); RIV, Flublok recombinant influenza vaccine (Sanofi, https://www.sanofi.com).

Twenty-four days after receiving a vaccine booster (LAIV and RIV), or 25 days after the second vaccine booster (IIV3), we inoculated ferrets with 10^6^ PFU of H5N1 MI/24 and observed them for clinical signs of disease. Mock-immunized ferrets showed signs of moderate disease as previously described (*24*), including sporadic nasal discharge, sneezing, mild lethargy, transient fever (average maximum temperature rise 2.2°C above baseline), and a maximum weight loss of 15.3% (95% CI 12.5%–18.1%) (Table 2). In contrast, ferrets immunized with LAIV displayed milder symptoms with less frequent nasal discharge and sneezing, characterized by a lower average maximum temperature rise and lethargy score, along with a maximum weight loss of 6.2% (95% CI 2.5%–9.9%; p = 0.0292). Those vaccinated with RIV and IIV3 also demonstrated limited protection and reduced frequency of nasal discharge and sneezing; although their maximum weight loss of 9.3% (95% CI 6.2%–12.3%) in the RIV group and 13.6% (95% CI 8.9%–18.2%) in the IIV3 group were not significantly less than the mock-immunized group (RIV, p = 0.083; IIV3, p = 0.84). Each group recovered significantly more weight than did mock-immunized groups after 7 DPI (on day 7, p = 7.9 × 10^−7^ for LAIV, p = 1.6 × 10^−7^ for RIV, and p = 0.006 for IIV3) (Figure 6, panel A).

**Table 2:** Pathogenicity of A(H5N1) influenza virus in naive and vaccinated ferrets in study of the effect of seasonal human influenza vaccination on pathogenesis of human influenza A(H5N1) clade 2.3.4.4b virus in the ferret model*

Vaccine group	Mean maximum weight loss, %	Mean temperature rise, °C	RII	Mean log_10_ peak titer
Mock	15.3 (12.5–18.1)	2.2 (2.0–2.4)	1.4	5.8 (5.4–6.2)
LAIV	6.2 (2.5–9.9)	1.2 (0.7–1.7)	1.0	4.5 (3.9–5.0)
RIV	9.3 (6.2–12.3)	1.2 (1.1–1.4)	1.1	5.6 (5.3–5.9)
IIV3	13.6 (8.9–18.2)	1.2 (1.0–1.4)	1.1	5.9 (5.5–6.3)

*Values in parentheses are 95% CIs. n = 6 ferrets per vaccine group, n = 12 ferrets in the mock group. The maximum values for each individual ferret were used to calculate maximum weight loss, temperature, and virus titer in nasal washes.IIV3, Fluarix vaccine (GlaxoSmithKline Biologicals, https://www.gsk.com); LAIV, FluMist vaccine (AstraZeneca, https://www.astrazeneca.com); RII, relative inactivity index; RIV, Flublok vaccine (Sanofi, https://www.sanofi.com).

### Vaccine Protection Against H5N1 Virus Replication and Shedding

Next, we evaluated the effect of seasonal influenza vaccination on viral replication and shedding after infection with H5N1 MI/24 virus in ferrets. As we demonstrated (Figure 6, panel B), ferrets in the mock group shed infectious virus above a GMT of 10^5.2^ PFU/mL through 4 DPI and maintained a GMT of ≈10^4.2^ PFU/mL at 6 DPI, whereas ferrets vaccinated with LAIV exhibited significantly lower level of infectious virus in the NW (4 DPI, p = 2× 10^−5^), with GMT of 10^3.4–4.2^ PFU/mL on 1–4 DPI, and cleared the virus by 6 DPI (Figure 6, panel B). Ferrets vaccinated with IIV3 and RIV shed comparable amounts of virus through 4 DPI; however, both groups displayed a major reduction in viral shedding by 6 DPI, with no detectable virus present by 8 DPI. Beyond virus shedding in the NW specimens, the MI/24 virus was capable of replicating in both the upper and lower respiratory tract, as well as extrapulmonary tissues such as the olfactory bulb and brain, which was consistent with previous findings (*24*). Compared with the mock group, all 3 vaccinated groups exhibited similar levels of viral replication in the ethmoid turbinate, soft palate, the olfactory bulb, and brain tissues at 3 DPI. Of note, ferrets in the LAIV group had significantly reduced infectious virus levels in the nasal turbinates (p = 0.004, relative to the mock-immunized group), trachea (p = 0.004), and lung (p = 0.043); only 1 of 3 LAIV-vaccinated ferrets had low levels of virus (10^1.8^ PFU/g) in the trachea, and none of the ferrets in this group showed detectable virus in the lung (Figure 6, panel C). In contrast, both IIV3 and RIV vaccination did not result in substantial effects on viral replication or detection frequency across respiratory tract and extrapulmonary tissues (Figure 6, panel C).

## Discussion

In this article, we demonstrate that immunization of naive ferrets with commercially available vaccines against human seasonal influenza confers some cross-protection against infection and disease caused by HPAI H5N1 virus. This evidence of cross-protection is consistent with previous observations that prior infection of ferrets with pH1N1 virus provides a degree of cross-protection against subsequent infection with HPAI H5N1 virus (*21*). Although human seasonal influenza vaccines confer protection against disease without the risks associated with natural influenza virus infection, the immunity that is induced in vaccinated persons is less potent and less cross-reactive than immunity induced by natural infection (*32*–*34*). Our findings indicate that despite minimal detectable cross-reactive anti-H5 responses, commercially available influenza vaccines can reduce disease severity in immunologically naive H5N1-infected ferrets.

Vaccination of naïve ferrets with LAIV, RIV, or IIV3 induced serologic responses against the vaccine antigens, as expected. No cross-reactive response against H5 was detected after vaccination (Figures 1, 3). H5 and H1 HAs are in the same phylogenetic group and cross-reactive antibodies that recognize both H1 and H5 were identified, which includes both antibodies that react with the relatively conserved stalk region of HA (*35*) and some that interact with the less conserved globular head (*36*–*38*). Both LAIV and RIV immunization did induce binding antibodies against the stalk region from H1 (Figure 3). Titers of those antibodies increased after H5N1 infection, whereas those against the globular head region of H1 and H3 waned, demonstrating that serologic cross-reactivity between H5 and H1 is mainly limited to the stalk region (Figure 3).

H5N1 viruses share an NA subtype with the pH1N1, and antibodies against N1 from both human H1N1 and HPAI H5N1 have been identified after infection with pH1N1 (*39*–*41*). Because of the shared NA subtype, substantial cross-reactive binding antibodies to N1 NA of H6N1 2.3.4.4b A have been detected in the human population (*5*; Z.-N. Li et al., unpub. data). Some ferrets vaccinated with LAIV and IIV3 did develop low but detectable cross-reactive binding antibodies against N1 from H5N1 clade 2.3.4.4b virus, and antibodies against N1 from pH1N1 virus were somewhat boosted by H5N1 infection (Figures 2, 4), again demonstrating cross-reactivity between these antigens. The RIV vaccine contains no NA and therefore did not induce any anti-NA response.

Despite the low cross-reactive response induced by the vaccines, vaccination still conferred some protection against H5N1 disease in ferrets. LAIV, which induced both antistalk antibodies and cross-reactive anti-N1 antibodies, gave the strongest protection against disease and reduced virus shedding. RIV, which induced antistalk HA antibodies but no anti-NA response, also provided some protection against weight loss and reduced the duration of virus shedding. IIV3, which induced only low levels of antistalk HA antibodies but did induce cross-reactive N1 antibodies, provided limited protection against weight loss and reduced the duration of virus shedding.

In addition, both LAIV and IIV3 were able to induce antibodies against NP, which is relatively conserved across different IAV subtypes, and NP antibodies might also contribute to cross-protection *42*). Cell-mediated immunity against influenza might be more broadly cross-protective against disease than antibodies because T-cell responses often target highly conserved internal proteins such as NP (*43*). Although both LAIV and IIV3 immunization induce T-cell responses, cell-mediated immune responses to inactivated vaccines are expected to be low and to target mainly the less conserved HA and NA, because NP and other internal proteins are only present at low and variable levels. The same is true for RIV, which does not include any influenza antigens other than HA.

Immunization with LAIV leads to some replication of the attenuated vaccine virus in the nasal cavity and induces localized inflammation and cytokine expression (*44*). Although we waited for 24 days after the last immunization before challenging ferrets with H5N1, it is possible that low-level localized inflammation might have contributed some nonspecific protection against infection. However, the IM-administered RIV vaccine also conferred major protection against disease, although its effect on virus shedding was more limited, suggesting that nonspecific local immunity alone was not required for cross-protection.

The findings that both prior human seasonal IAV infection and vaccination with commercially available human seasonal influenza vaccines confers some cross-protection against disease caused by the B3.13 genotype H5N1 in the ferret model is intriguing. However, unlike most adult humans who receive seasonal influenza vaccination, the laboratory ferrets used in this study are completely naive to influenza, and their response to the vaccines might not fully reflect the adult human response. The function and extent of cross-protection between immunity to human seasonal viruses and potentially zoonotic HPAI requires further investigation, particularly given the complex landscape of existing IAV immunity in the general population and its potential influence on the immune response elicited by seasonal influenza vaccination. This consideration is especially true for LAIV. Despite the cross-protection against the H5N1 virus observed in LAIV-vaccinated ferrets, the ability of LAIV to replicate in the upper respiratory tract of humans with existing immunity and to elicit antibody response is limited (*45*).

In conclusion, this study demonstrates that seasonal influenza vaccination can potentially provide cross-protection against the B3.13 genotype of H5N1 virus. Although the applicability to humans is currently unknown, revealing this benefit in the naive ferret model is a crucial step to further exploring the benefits of seasonal influenza vaccination in reducing the effects of HPAI H5N1 in human populations.

Appendix Additional information about effects of seasonal influenza vaccines on influenza A(H5N1) clade 2.3.4.4b virus infection in ferrets.
