# Supplementary material for: Effect of Seasonal Influenza Vaccines on Avian Influenza A(H5N1) Clade 2.3.4.4b Virus Infection in Ferrets
Source: Emerg Infect Dis. 2025 Oct;31(10):1950–60. doi: 10.3201/eid3110.250668 (PMC12483109; doi:10.3201/eid3110.250668)
Supplement: Appendix — Additional information about effects of seasonal influenza vaccines on influenza A(H5N1) clade 2.3.4.4b virus infection in ferrets. [file 25-0668-Techapp-s1.pdf]

Article DOI: https://doi.org/10.3201/eid3110.250668

EID cannot ensure accessibility for supplementary materials supplied by authors. Readers who have difficulty accessing supplementary content should contact the authors for assistance.

Effect of Seasonal Influenza Vaccines on Influenza A(H5N1) Clade 2.3.4.4b Virus Infection in Ferrets

Appendix

Appendix Table. Recombinant antigens included in the MIADA assay*

Antigen category	Antigen abbreviation	Virus strain	Type (subtype or lineage)	Influenza HA group	Resource	GISAID Accession No.
Globular head HA1	ID/18	A/Idaho/07/2018	A(H1N1)pdm09	1	CDC	EPI1206974
Globular head HA1	WI/19	A/Wisconsin/588/2019	A(H1N1)pdm09	1	CDC	EPI1661758
Globular head HA1	Syd/21	A/Sydney/5/2021	A(H1N1)pdm09	1	CDC	EPI1957293
Globular head HA1	WI/22	A/Wisconsin/67/2022	A(H1N1)pdm09	1	CDC	EPI2224788
Globular head HA1	Cam/20	A/Cambodia/e0826360/2020	A(H3N2)	2	CDC	EPI1837753
Globular head HA1	Dar/21	A/Darwin/6/2021	A(H3N2)	2	CDC	EPI2415902
Globular head HA1	MA/22	A/Massachusetts/18/2022	A(H3N2)	2	CDC	EPI2096148
Globular head HA1	Ind/05	A/Indonesia/5/2005	A(H5N1)	1	CDC	EPI376537
Globular head HA1	TX/24	A/Texas/37/2024	A(H5N1)	1	CDC	EPI3171488
Globular head HA1	Ind/05	A/Indonesia/5/2005	A(H5N1)	1	CDC	EPI376537
Globular head HA1	AW/21	A/American Wigeon/South Carolina/22-000345-001/2021	A(H5N1)	1	CDC	EPI1985910
Globular head HA1	TX/24	A/Texas/37/2024	A(H5N1)	1	CDC	EPI3171488
Ectodomain	TX/24	A/Texas/37/2024	A(H5N1)	1	CDC	EPI3171488
Globular head HA1	WA/19	B/Washington/02/2019	Victoria lineage	NA	CDC	EPI1368874
Globular head HA1	Aus/21	B/Austria/1359417/2021	Victoria lineage	NA	CDC	EPI1845793
Globular head HA1	Phu/13	B/Phuket/3073/2013	Yamagata lineage	NA	CDC	EPI529345
N1 NA	BR/18	A/Brisbane/02/2018	A(H1N1)pdm09	NA	Sino Biological†	EPI1799927
N1 NA	WI/19	A/Wisconsin/588/2019	A(H1N1)pdm09	NA	CDC	EPI1661757
N1 NA	Syd/21	A/Sydney/5/2021	A(H1N1)pdm09	NA	CDC	EPI1957292
N1 NA	WI/22	A/Wisconsin/67/2022	A(H1N1)pdm09	NA	CDC	EPI2224787
N2 NA	Cam/20	A/Cambodia/e0826360/2020	A(H3N2)	NA	Sino Biological	EPI1837753
N2 NA	Dar/21	A/Darwin/6/2021	A(H3N2)	NA	CDC	EPI2415900
N2 NA	MA/22	A/Massachusetts/18/2022	A(H3N2)	NA	CDC	EPI2096147
N1 NA	AW/21	A/American Wigeon/South Carolina/22-000345-001/2021	A(H5N1)	NA	CDC	EPI1985912
N1 NA	TX/24	A/Texas/37/2024	A(H5N1)	NA	CDC	EPI3171486

Antigen category	Antigen abbreviation	Virus strain	Type (subtype or lineage)	Influenza HA group	Resource	GISAID Accession No.
HA stalk	MI/15	A/Michigan/45/2015	A(H1N1)pdm09	1	CDC	EPI662594
HA stalk	Sin/16	A/Singapore/INFIMH-16–0019/2016	A(H3N2)	2	CDC	EPI780183
IAV NP	BR/07	A/Brisbane/10/2007	A(H3N2)	NA	CDC	EPI353307

*CDC, Centers for Disease Control and Prevention; NA, not applicable
†Sino Biological US Inc, Pennsylvania, PA

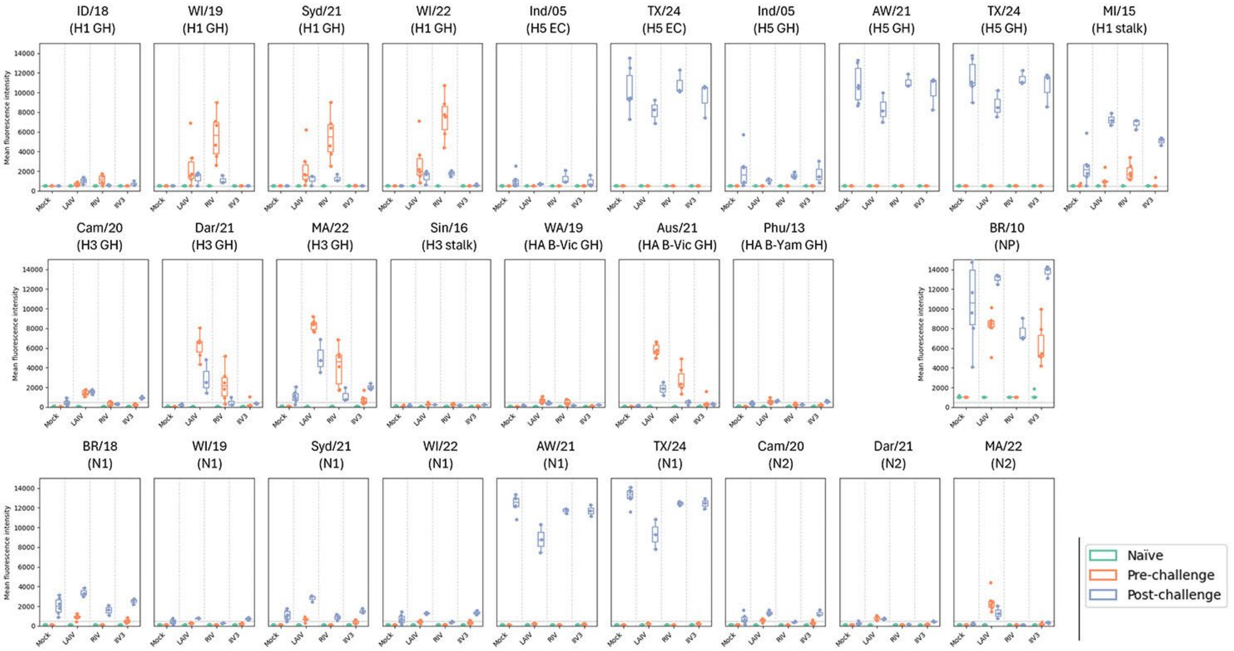

Appendix Figure. Ferrets were immunized with LAIV or RIV twice (n=6 ferrets per group), 3 weeks apart, or with IIV3 (n=6) three times, at 3-week intervals, or were mock immunized (n=12). Twenty-four or 25 days after the final immunization, ferrets were challenged with influenza A/Michigan/90/2024(H5N1), a 2.3.4.4b clade virus. Blood samples were collected before immunization ("Naïve"), on the day of challenge ("Pre-challenge"), or approximately 3 weeks post-infection ("Post-challenge"). MIADA assays were performed as described in the Methods, using a panel of antigens including hemagglutinin (HA) from influenza A(H1N1)pdm09, A(H3N2), and A(H5N1) viruses and from influenza B viruses, and neuraminidase (NA) from A(H1N1)pdm09, A(H3N2), and A(H5N1) viruses, as well as influenza A nucleoprotein. See Appendix Table for antigen details.
